# PRIMO: An Interactive Homology Modeling Pipeline

**DOI:** 10.1371/journal.pone.0166698

**Published:** 2016-11-17

**Authors:** Rowan Hatherley, David K. Brown, Michael Glenister, Özlem Tastan Bishop

**Affiliations:** Research Unit in Bioinformatics (RUBi), Department of Biochemistry and Microbiology, Rhodes University, Grahamstown, 6140, South Africa; Universita degli Studi di Padova, ITALY

## Abstract

The development of automated servers to predict the three-dimensional structure of proteins has seen much progress over the years. These servers make calculations simpler, but largely exclude users from the process. In this study, we present the PRotein Interactive MOdeling (PRIMO) pipeline for homology modeling of protein monomers. The pipeline eases the multi-step modeling process, and reduces the workload required by the user, while still allowing engagement from the user during every step. Default parameters are given for each step, which can either be modified or supplemented with additional external input. PRIMO has been designed for users of varying levels of experience with homology modeling. The pipeline incorporates a user-friendly interface that makes it easy to alter parameters used during modeling. During each stage of the modeling process, the site provides suggestions for novice users to improve the quality of their models. PRIMO provides functionality that allows users to also model ligands and ions in complex with their protein targets. Herein, we assess the accuracy of the fully automated capabilities of the server, including a comparative analysis of the available alignment programs, as well as of the refinement levels used during modeling. The tests presented here demonstrate the reliability of the PRIMO server when producing a large number of protein models. While PRIMO does focus on user involvement in the homology modeling process, the results indicate that in the presence of suitable templates, good quality models can be produced even without user intervention. This gives an idea of the base level accuracy of PRIMO, which users can improve upon by adjusting parameters in their modeling runs. The accuracy of PRIMO’s automated scripts is being continuously evaluated by the CAMEO (Continuous Automated Model EvaluatiOn) project. The PRIMO site is free for non-commercial use and can be accessed at https://primo.rubi.ru.ac.za/.

## Introduction

Studying the three-dimensional (3D) structure of a protein is crucial to gaining insights into its function, which is one of the driving principles behind structural biology [1] and structural bioinformatics [2]. Experimental techniques, such as X-ray crystallography and NMR provide an accurate means to determine the structure of a protein [3]; and cryo-electron microscopy techniques have achieved atomic resolution [1]. The speed of these techniques however, falls far behind that of sequencing technologies, leaving a large gap between the known sequences of proteins and their structures [4].

In the absence of experimental data, *in silico* prediction of protein structures has become an important means of studying proteins in spite of this sequence-structure gap [5]. There are two broad approaches for achieving this, 1) template-based modeling (homology modeling and threading) and 2) template-free modeling or *ab initio* techniques [6–8]. In the presence of a suitable template, homology modeling provides an accurate way to determine the 3D structure of a protein with many successful applications [7,9–11].

Homology modeling involves predicting the structure of a protein using homologous protein(s), with known structure(s), as template(s). This approach works due to the principle that the structure of a protein is far more conserved than its primary amino acid sequence [12]. The most well-established and widely used program for this is MODELLER [13].

While the software for MODELLER can be downloaded and used locally for protein structure prediction, there are many users with little to no experience in structural bioinformatics who rather turn to one of many automated modeling servers. Examples of these include the automated server, ModWeb [14], SWISS-MODEL [15] and Phyre2 [16].

ModWeb is a modeling server that forms part of ModBase [14]. It is a fully automated server that performs modeling using MODELLER. There are several options that can be selected that determine how templates are identified as well as what criteria the server will use to select a best scoring model. SWISS-MODEL [15] is one of the most widely-used and longest standing servers which uses its own comparative modeling functions. The server can perform fully automated modeling, but does also allow users to select templates for modeling. The server also has functionality to model ligands into structures, as well as to incorporate quaternary structure into modeling, if this is known from the template. Phyre2 [16] is an automated modeling server that is widely used and models with an accuracy comparable to other top modeling servers. The server provides two fully automated options (normal and intensive mode) which uses its sophisticated comparative modeling algorithms, combined with other tools such as Poing [17] to perform *ab initio* modeling and 3DLigandSite [18] to predict ligand binding sites.

While automated servers are useful, they do exclude users from the modeling process. This lack of engagement limits their understanding of what is happening in the background, making it difficult to critically evaluate their models, as well as make alterations and improvements. An example of a server which does allow for this is the HHpred homology detection server [19], which provides an interface that links its own template identification algorithms to MODELLER to generate a 3D structure for further analysis. The HHpred server is one of the most flexible one by allowing users to select templates and modify their alignments. However, it does not allow users to modify modeling parameters, such as number of models to be produced or refinement level; both of which can improve model quality. It also does not trim its alignment at the N- and C-termini to only include sections covered by the template. This causes MODELLER to attempt to model these regions without template information, producing indistinct loops in these regions.

Apart from homology modeling servers, some other techniques and servers have also been developed which work well especially on more challenging protein targets, i.e. those with no homologous templates of known structure. The most successful of these servers is probably I-TASSER [20] which incorporates a combination of threading, fragment assembly and *ab initio* techniques as part of its template-based modeling protocol. While this server is well suited to challenging targets it is not ideal for more standard modeling jobs as it can be time consuming, often limiting users to a single modeling run at a time, spanning over a number of days.

Considering all the above points, we have developed the PRotein Interactive MOdeling (PRIMO) pipeline to provide a user-inclusive online modeling resource. It incorporates a user-friendly interface that has been designed to guide users through each stage in the homology modeling process. Keeping novice users in mind, the interface is simple and easy to learn, while allowing more experienced users to alter parameters and exhibit control over their modeling jobs. Multiple options are provided for both template identification and template-target sequence alignment. Additionally, PRIMO allows users to alter parameters specific to MODELLER, such as refinement level and number of models produced, as well as allowing users to model specific ligands and ions found within template PDB files.

PRIMO is being developed as part of H3ABioNet [21] for use by the H3Africa Consortium [22]. Research groups around Africa, as part of the Consortium, have been sequencing a large number of human genomes linked to various diseases and identify disease associated novel SNPs. PRIMO can be used to analyze disease related proteins and relevant nonsynonymous SNPs. In this way, PRIMO can help to advance the progress towards the Consortium’s scientific goals. However, the usage of PRIMO goes beyond the Consortium’s targets as it is designed to model proteins from any organism.

Here we describe the features of the PRIMO web interface and assess the backend scripts of PRIMO to demonstrate the accuracy of the pipeline when choosing fully automated options for modeling protein targets of interest.

## Methods

**Fig 1:**
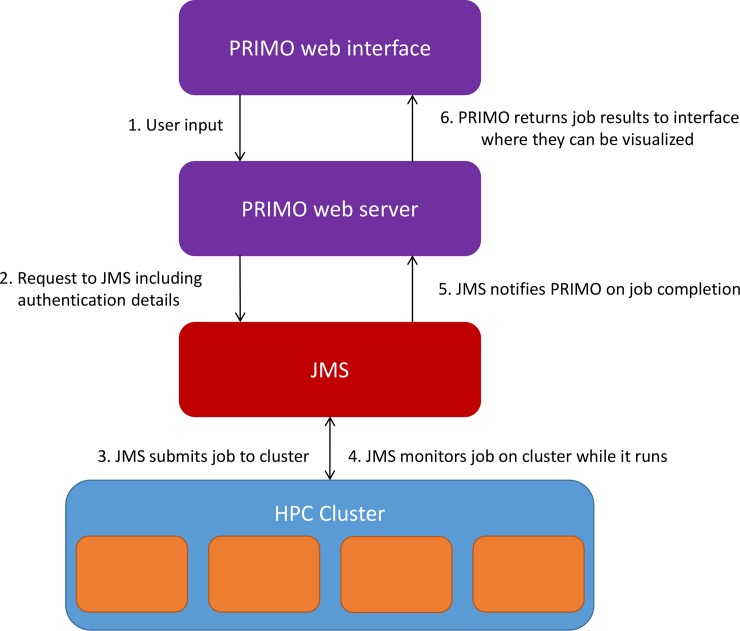
Submitting jobs via JMS. The figure illustrates the process of submitting a job to the cluster via JMS.

The backend functionality of PRIMO was written in Python, presented as three separate tools in a local version of the Job Management System (JMS) [23]. The PRIMO pipeline currently provides options which use HHsuite [24], protein BLAST [25] Clustal Omega [26], MAFFT [27], MUSCLE [28], T-Coffee [29], MODELLER [13] and PROCHECK [30]. The PRIMO web interface is written as a single page web application, managed using the Django web framework. Communication between the PRIMO web interface and the PRIMO tools is managed through AJAX calls via the JMS API. The diagram presented in Fig 1 illustrates the process by which jobs are submitted from PRIMO to the cluster via JMS. When a user submits a modeling job from the PRIMO interface, their input parameters are sent to the PRIMO server. PRIMO then compiles these parameters into a request to be sent to JMS. Authentication details for JMS are also added to the request at this point. Once the request has been compiled, it is sent to JMS, which submits the job to the cluster and returns the job ID to the PRIMO web server. PRIMO then simply returns a message to the interface that the job was submitted successfully, while JMS monitors the job on the cluster. When the job finishes running on the cluster, JMS notifies PRIMO that the results are available. PRIMO then collects the results and returns them to the interface, where the user can interact with them.

### PRIMO modeling algorithm

**Fig 2:**
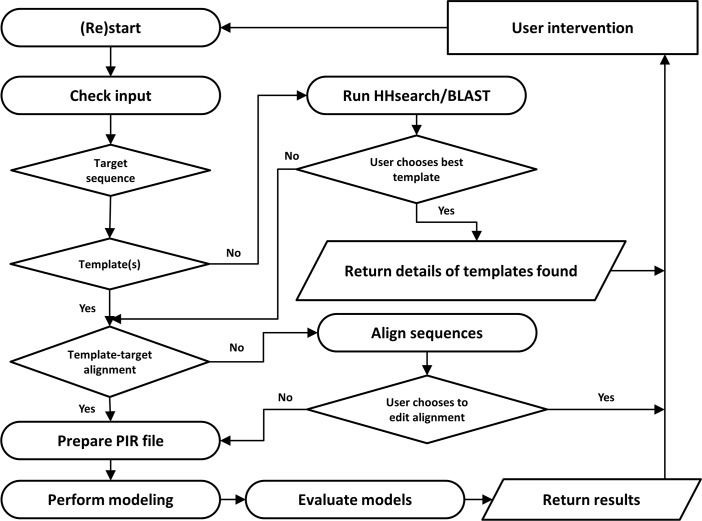
Flow chart depicting the modeling algorithm incorporated by PRIMO. The steps involved in modeling using PRIMO can be seen as an interactive process in which the user can supply and edit input as they see fit, while PRIMO chains these steps together to model protein targets of interest.

The PRIMO modeling algorithm is displayed in Fig 2. The minimum input required for the server is the sequence of a protein (target protein) to be modeled. Thereafter, the process is divided into three steps: 1) template identification and selection, 2) target-template sequence alignment and 3) modeling and model evaluation. Each step follows on to the next and allows for user inspection and input between these stages.

#### Template identification

This step involves helping the user find suitable templates for modeling. PRIMO allows users to select either HHsearch of HHsuite or protein BLAST to search for templates. BLAST is set as a default search option as it runs substantially faster than HHsearch, and identifies closely related templates if any are present in the PDB. A local version of BLAST is used to query the target sequence against a National Center for Biotechnology Information (NCBI) database of PDB files downloaded from ftp://ftp.ncbi.nlm.nih.gov/blast/db. Output from BLAST is parsed to extract information about each template, including the PDB ID and chain, template-target sequence identity, query coverage and the alignment produced when running BLAST. Alternatively, HHsearch can be run if distant homologs need to be identified. This option incorporates various programs from the HHsuite package. HHblitz is run to search the target sequence against the HHsuite uniprot20 database. Secondary structure is added to the A3M alignment, an alignment format generated by HHblitz and used by HHsearch, before converting it to hidden Markov model using HHmake. HHsearch is then used to search against the HHsuite pdb70 database to identify templates. The resulting hhr file is parsed to extract the same information obtained if BLAST was run.

#### Target-template sequence alignment

For each template selected, the PDB file is parsed to extract its sequence. Both missing residues and non-standard amino acids are replaced with an “X” character, so this information may be included in the alignment. PRIMO performs the alignment using MAFFT, MUSCLE, Clustal Omega or T-Coffee. The template-target alignment provided by protein BLAST or HHsearch may also be used if one of these was run for template identification.

#### Modeling and model evaluation

The final step in the modeling process involves using the target-template sequence alignment and the template PDB file(s) to generate a PIR file and modeling script. The alignment undergoes some preprocessing before being converted to PIR format. Primarily this involves replacing the missing residue characters with gap characters (“-“) and modified residues with period (“.”) characters, since this is how MODELLER recognizes modified residues. The sequences also undergo trimming at each end to ensure that the parts of the target sequence being modeled have a corresponding template section at each end. Finally, each template sequence is checked against the sequence extracted from its PDB file to ensure that it is correct. The starting and ending residues in each template, which is required by MODELLER is also determined, and added to the PIR file. The PIR file is required by MODELLER to link the template-target alignment to the specific segments of each template PDB file used in modeling. Once the PIR file has been created, the modeling script is prepared, then run using MODELLER. After modeling has completed, the models are evaluated by MODELLER’s normalized DOPE function (DOPE Z-score) [31], as well as PROCHECK.

If ligands are specified for modeling, an additional set of steps is taken to prepare the PIR file before modeling can begin. In this context, “ligands” include any HETATM record found in the template PDB, excluding non-standard amino acids; for example substrates, ions, inhibitors and solvent molecules. All ligands specified are identified within their respective template PDB file. The position of the ligand that occurs last in the coordinate section is noted and becomes the ending residue for that template in the PIR file. All residues and ligands that occur up to this position are then appended to the template entry in the original PIR file. In the target sequence, gap characters are added to match the length of the template. Gaps are then replaced with period characters to match the positions of ligand molecules of interest that occur within the template, since MODELLER recognizes these characters as ligands as well. In addition to PIR file modifications, additional parameters are given to the modeling script to instruct MODELLER to read in ligands or solvent molecules where applicable.

### PV-MSA: a JavaScript wrapper combining the functionality of PV and MSA

PV [32] is a widely used JavaScript plugin for 3D protein visualization. Similarly, BioJS MSA (http://msa.biojs.net/) is a JavaScript component used to visualize multiple sequence alignments. Although useful in their own right, the need to view a structure in conjunction with its sequence often arises in bioinformatics. In addition, these tools can be difficult to use as their application programming interfaces (API) are fairly unintuitive. To cater for this, we have developed PV-MSA, a wrapper that combines the functionality of PV and MSA in a single JavaScript plugin. PV-MSA also provides a simplified API that makes a fair amount of the functionality of both PV and MSA available. For functionality that has not been included yet, PV-MSA provides direct access to the underlying PV and MSA objects.

Over and above simply wrapping the two plugins, PV-MSA links the selection functionality. For example, a user can select a residue in the protein structure and it will automatically be highlighted in the alignment. The alignment is automatically scrolled to the selected position. Similarly, if a residue is selected in the alignment, its location is highlighted on the corresponding structure. PV-MSA also allows structures to be superposed. In such cases, selecting a residue in one structure will also highlight the aligned residue in the superposed structure. This selection is based on the alignment, and as such, gaps and missing residues are taken into account.

Multiple structures and their sequences can be loaded into the plugin at once and structures and sequences can be hidden and shown independently. PV-MSA also allows users to visualize and select ligands and ions in a structure. Selecting a ligand displays a label over the ligand with the ligand name. Functionality has also been included to resize both the PV and MSA plugins together and independently as the user needs. The PV-MSA plugin can be downloaded from https://github.com/davidbrownza/PV-MSA.

### Testing of PRIMO scripts

**Fig 3:**
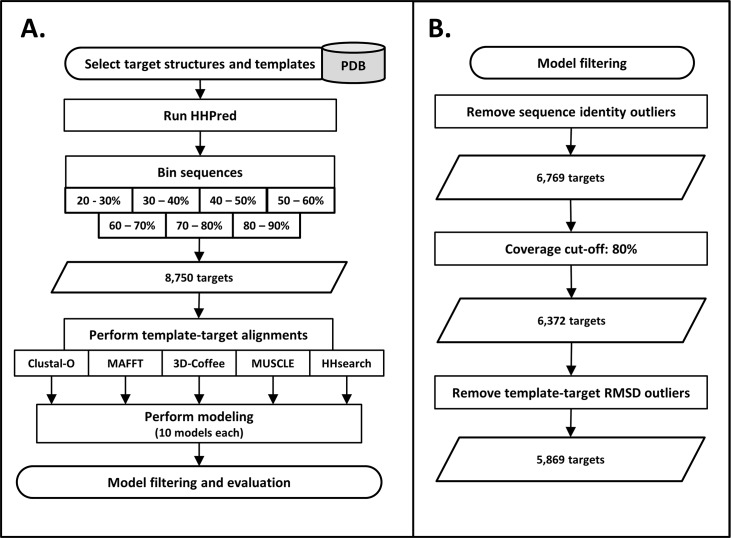
Workflow followed to test the PRIMO backend scripts. A) Overview of the steps followed when modeling known protein targets from the PDB. B) Filtering steps involved to reach the final 5,869 targets.

In order to evaluate the performance and reliability of the PRIMO modeling scripts, tests were run which involved modeling target proteins from the PDB with known structures. The process followed to test the PRIMO scripts is shown in Fig 3A. Target structures were fetched at random from the PDB and templates for modeling were identified using PRIMO’s template identification protocol (see above). The template-target sequence identity values were recorded for each set and target-template combinations were binned according to this. This process was repeated until there were 1250 different structures in each bin. The bin ranges used in this manuscript include only the lower value shown (i.e. a template with 30% sequence identity was included in the 30–40% bin, not the 20–30% bin).

For each entry in each bin, the targets were aligned to the templates using the four sequence alignment programs provided by PRIMO, as well as the HHsearch alignment calculated during template identification.

For MAFFT, a script was written to mimic the MAFFT-homologs alignment option. This firstly runs a local version of protein BLAST [25] on both the template and the target to retrieve 50 closely-related sequences to each, before combining these two sets of sequences and aligning them using MAFFT. Similarly, for the T-Coffee alignment, a script was written to mimic the functionality of Expresso [33]. Expresso makes use of 3D-Coffee [34], which incorporates structural information when running T-Coffee. While Expresso runs BLAST to identify homologous PDB structures as input for 3D-Coffee, our mimic script runs 3D-Coffee using the alternative templates identified during template identification (excluding the target PDB). These modifications were made because each of these programs requires calls to external webservers, which slow down substantially and eventually crash when running thousands of modeling jobs.

For each alignment produced, modeling jobs were run using MODELLER, producing 10 models per run using very slow refinement. Due to sequence trimming of the PIR preparation step, not all models from the same target-template set were the same size when modeled using different alignment options. To normalize the models, the PDB files in each modeling set were trimmed to the longest common segment of all models in that set.

Models also went through a series of filtering steps (Fig 3B). After performing target template alignments using the different alignment programs, some models fell outside their designated sequence identity bin (see S1 Fig). This is because sequence identity is calculated from the alignment between target and template, so realigning with different programs produced different results. To make the modeling sets comparable, only sets where the template-target alignment from all five alignment programs fell in the same bin were included. The target coverage was also calculated for each modeling set. Here, sets were only included if at least 80% of the target sequence was modeled, for all five alignment options. The final filtering step involved calculating the RMSD between each template and target PDB file using BioPython, divided into their respective bins. Outliers were calculated and removed from each of these sets. This was done to account for target-template combinations with large conformational differences, where RMSD could not be used to assess the modeling accuracy.

After filtering, the models were evaluated. DOPE Z-score calculations were performed on each model produced, to select the top model from each set. The top model and the target PDB structure were then compared by calculating RMSD, Global distance test–high accuracy (GDT-HA) score, template modeling (TM) score [35] and Local distance difference test (lDDT) score [36]. Both GDT-HA and TM score values were calculated using TMscore software downloaded from the Zhang Lab (http://zhanglab.ccmb.med.umich.edu/TM-score/). Software to calculate lDDT score was downloaded from http://swissmodel.expasy.org/lddt.

A PDB remodel set was also produced and evaluated for each target. Each of the targets was modeled using its own PDB structure as a template, representing ideal modeling conditions and giving an indication of the error produced by MODELLER itself.

### Testing model refinement options

In addition to testing the different alignment options provided by PRIMO, some tests were performed to evaluate the different refinement options provided when modeling using MODELLER. These were done using the MAFFT modeling set, with the same PIR files as calculated for the MAFFT alignments. These were also only done using the final set of models evaluated after filtration steps were carried out. The only parameter altered was the refinement level option in the modeling script. The additional refinement levels tested included none and fast. These were compared with the very slow option used in the alignment studies. Models were evaluated by DOPE Z-score and RMSD, as in the other tests.

### Modeling case studies

In order to demonstrate the performance of PRIMO when compared to other modeling options, two case studies were performed. These included the modeling of heat shock protein 70-x from *Plasmodium falciparum* (PfHsp70-x; accession: PF3D7_0831700) as monomer and X-linked tyrosine kinase from *Homo sapiens* (HsTXK; Accession: AAA74557.1), modeling with ligands. Online modeling servers tested included SWISS-MODEL [15], Phyre2 [16], ModWeb [14], HHpred [19] and I-TASSER [20]. SWISS-MODEL was run, allowing the server to select the best templates and build models without user intervention. Phyre2 was run using its intensive modeling mode. ModWeb was run using the very slow fold assignment option, but otherwise using default parameters. I-TASSER was run using its default parameters. HHpred was allowed to automatically select the top template and perform its alignment, but the alignment was manually trimmed in the PIR file at the N- and C-termini. For PfHsp70-x, four modeling sets were chosen from PRIMO; 1) Using the templates 5e84, 4jne and 5pfn, aligned using MAFFT with no further intervention; 2) The same template combination used in (1) except aligned using 3D-Coffee and no further intervention; 3) The same parameters used in (2), except with small manual edits to the alignment; 4) Using the templates 5e84, 5pfn and 3d2f and MAFFT as the alignment program–here only the final 80 residues of template 3d2f were used to model the C-terminal alpha-helical region of the protein to produce a longer and more complete model. For HsTXK, two modeling sets were chosen for PRIMO; 1) Using only template 4ot5, which comprises the catalytic domain of the kinase. This template structure was in complex with an inhibitor, 4-tert-Butyl-N-(3-{8-[4-(4-methyl-piperazine-1-carbonyl)-phenylamino]-imidazo[1,2-a]pyrazin-6-yl}-phenyl)-benzamide, (PDB ID: 481), which was also selected for modeling; 2) Template 1opk with its inhibitor, 6-(2,6-Dichlorophenyl)-2-{[3-(Hydroxymethyl)Phenyl]Amino}- 8-Methylpyrido[2,3-D]Pyrimidin-7(8h)-One, (PDB ID: P16). Both were aligned using 3D-Coffee with only minor manual edits to the alignment. All models were assessed using ProSA [37], Verify3D [38,39], PROCHECK [30], the QMEAN server [40] and DOPE Z-score [31].

### Independent assessment of PRIMO by CAMEO

As an additional validation step, PRIMO has been registered to participate in the CAMEO (Continuous Automated Model EvaluatiOn) project [4]. CAMEO provides modeling servers with the sequences of PDB structures that have yet to be released, which these servers must predict the structure of and return to CAMEO for independent evaluation. PRIMO has been registered with four different modeling options, which include using the different combinations of BLAST and HHsearch for template identification, and Clustal Omega and 3D-Coffee for sequence alignment (registered as PRIMO_BLAST_CL, PRIMO_BLAST_3D, PRIMO_HHS_CL and PRIMO_HHS_3D, respectively). Results of evaluation by CAMEO are displayed at http://cameo3d.org/.

## Results and Discussion

### PRIMO web interface

**Fig 4:**
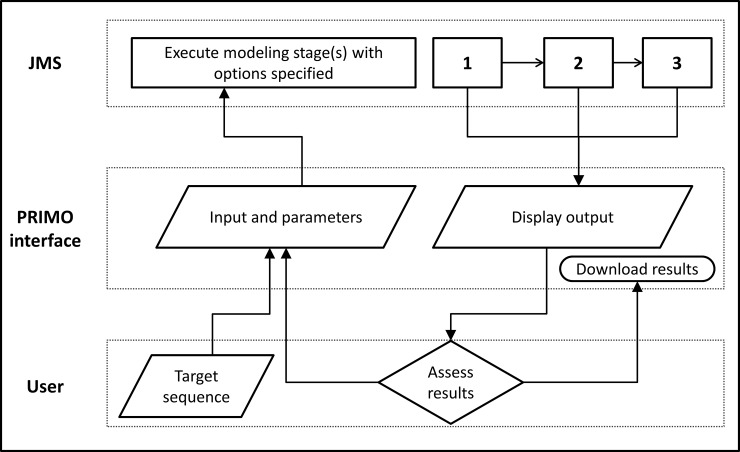
Architecture of PRIMO. The diagram shows how PRIMO can be separated into frontend web interface, which facilitates communication between the users and the backend scripts saved in the JMS, which perform the steps involved in homology modeling, which are numbered as 1) template identification, 2) template-target sequence alignment and 3) model building and evaluation.

The PRIMO website acts as a frontend to link users to the modeling scripts integrated into the JMS (Fig 4). The initial job overview page allows users to specify input and options for all three stages. PRIMO encourages a more ‘hands on’ approach to modeling, so users can go step-by-step through the process.

#### Input page

This provides an overview of the modeling job. Users can simply enter in a target sequence and begin the modeling process. PRIMO utilizes MODELLER [13], so users must also supply a MODELLER key. If no other input is provided, PRIMO will run using the default parameters set for each modeling stage. Alternatively, the page allows users to adjust the parameters for template identification, sequence alignment and modeling. For template identification, users can choose to search for templates using HHsearch [19] or protein BLAST [25], or specify templates themselves. They may also select one of five sequence alignment options available, which currently include MAFFT [27], MUSCLE [28], T-Coffee [29] and Clustal-Omega [26], as well as the alignment created by either HHsearch or BLAST. Modeling parameters can also be specified before the modeling job is started. Thereafter, the PRIMO interface guides users through each step in the homology modeling process. Input for each stage is processed and submitted to our local cluster, utilizing the JMS [23].

#### Template identification

If automatic template identification is run, the templates identified are displayed, including information about sequence identity and query coverage. Templates can be selected through simple check boxes to be included in the target-template alignment stage. Users can also click on the ID of any template, which links directly to its entry in the PDB, allowing users to further assess the quality of each template. The templates can be individually selected and displayed to assess their conformations for multiple-template modeling. The alignment produced by HHsearch or BLAST (whichever was run) is also displayed in order for the user to assess the suitability of each template as well as inspect query coverage. The interface also provides options to allow the modeling of ligands found within any of the templates. A drop down list appears for each template returned, which details ligands that can be included in the modeling run.

#### Target-template alignment

Sequences are extracted from the templates and aligned to the target sequence, using the alignment option selected. The alignment is displayed in an integrated alignment viewer and can be inspected and edited manually by the user before moving on to the modeling stage. The alignment editor validates changes that the user makes in order to prevent edits, which would cause the modeling stage to fail. In template sequences, gaps can be added anywhere, but the sequences can only be trimmed from the outsides. If the edited sequence cannot be found within the original sequence (excluding gaps), it is invalid. The target sequence can be edited in just about anyway, so long as valid characters for amino acids and gaps (‘-‘) are used.

#### Model building and evaluation

**Fig 5:**
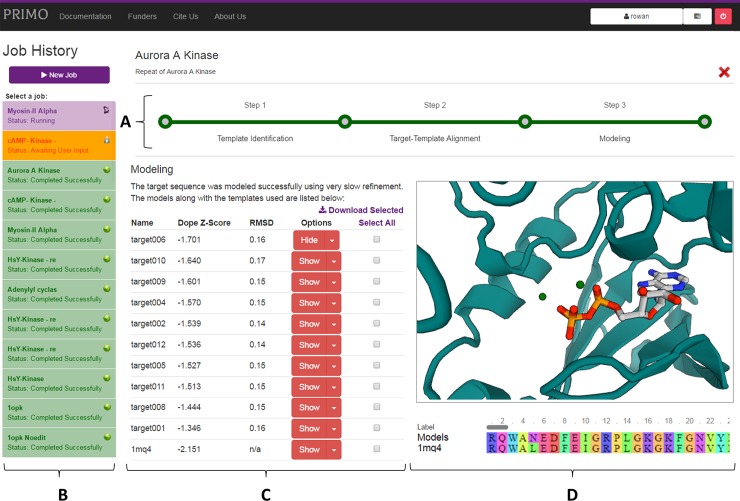
Modeling results page. As with other stages, the progress bar (A) and job history set (B) are displayed on the page. These allow navigation to within the current jobs and between different modeling jobs, respectively. Completed jobs are shown in green, those awaiting user input in yellow and running jobs in purple. The list of models are tabulated (C), ranked by their DOPE Z-scores. This table can be used to select and download models produced, link to their evaluation page, as well as show them in the interactive protein viewer (D). In the viewer is the top model (teal), zoomed in to show ADP and Mg ions that have also been modeled from the template.

The sequence alignment is utilized to prepare a PIR file, which is used by MODELLER. Modeling is performed using the parameters specified in the input page and the models are assessed by DOPE Z-score calculations. The top models are listed and can be visualized using the integrated PV-MSA PDB viewer provided (Fig 5). Additionally, each model contains a drop down link to the evaluation page. This displays plots produced by PROCHECK, which includes a Ramachandran plot, as well as nine other plots which describe the stereochemical quality of the model. The page also provides links to various other model evaluation sites. Currently this includes the ProSA [37] QMEAN [40] and Verify3D [38,39] servers.

#### Job history

Jobs are linked to the users’ accounts, which comprise an instant sign-in. Users can navigate to previous jobs run, as well as to different stages in their current jobs, alter parameters and rerun jobs. Email notifications can also be turned on to notify users when a job is complete or requires attention.

### Submitting jobs to the cluster via JMS

PRIMO makes use of a unique system to submit jobs to the underlying cluster (Fig 1). JMS [23] has been developed as a web-based workflow management system and cluster front-end for high performance computing (HPC). It is able to store custom tools and scripts, and manage their execution on an HPC cluster. The reason that JMS is used for submitting jobs is that it abstracts away the complexity of managing the job on the cluster and drastically reduces the time taken to develop the PRIMO web server. PRIMO was originally developed as a series of command-line scripts. We were able to upload these scripts to JMS directly via the JMS web interface. After that, building the PRIMO website simply involved building a custom interface that interacted with the JMS web API. Submitting and managing the job on the cluster is handled entirely by JMS while the PRIMO web server merely has to wait for a notification from JMS that the job has completed.

### Accuracy of the PRIMO backend scripts

While the PRIMO website was designed to promote user involvement during each step in the homology modeling process, the backend scripts are capable of performing fully automated modeling. Here we present the accuracy of PRIMO, when no user intervention occurs during the modeling process.

To assess the tools and algorithms incorporated into PRIMO, an evaluation study was performed by modeling proteins with known structure from the PDB, using templates ranging from 20% to 90% sequence identity, as well as using five different alignment approaches. After modeling and filtering as explained in the Methods section, the final set included 5 869 modeled targets, comprising 293 450 models, to be evaluated.

**Fig 6:**
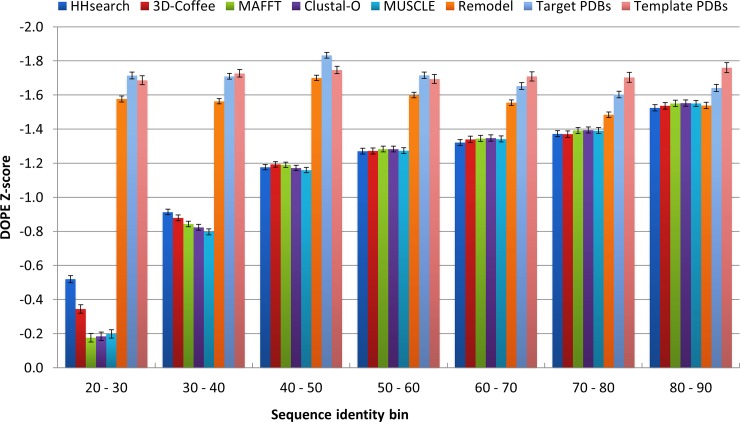
DOPE Z-score results obtained from testing the backend scripts of PRIMO. Modeling sets are divided into their respective bins and alignment programs used, as shown in the key. The ‘Remodel’ column depicts the scores for the targets modeled using themselves as templates. The ‘Target’ and ‘Template’ columns show the DOPE Z-scores of the known structures of the targets to be modeled and the templates used for modeling in each bin.

Due to the scale of the models produced, evaluations were performed using MODELLER’s DOPE Z-score, the results of which are shown in Fig 6. When evaluating models by DOPE Z-score, the desired value are -1.0 or below, as these models are considered native-like [41]. When testing the PRIMO scripts, models from 40–50% bin and above were on average below this cutoff. This is expected, as structures that share at least 40% sequence identity generally have similar structures [6]. The bins below 40% sequence identity displayed lower quality results, and alignments based on programs that use structural information, such as HHsearch and 3D-Coffee, outperformed the other alignment options, especially in the 20–30% sequence identity bin. This was also an expected result, since the addition of structural information is known to improve alignment accuracy in the case of low sequence identity [42].

The PDB structures of both the template and target PDBs were included in the DOPE Z-score evaluations, in order to get an idea of the quality of these structures. Similarly, each target was remodeled using itself as a template to represent modeling under ideal conditions (100% target-template sequence identity). These remodeled targets never matched the quality scores of either the templates or target PDB files. The models in the 80–90% sequence identity bin were the only set that on average matched the quality of the remodeled targets.

**Fig 7:**
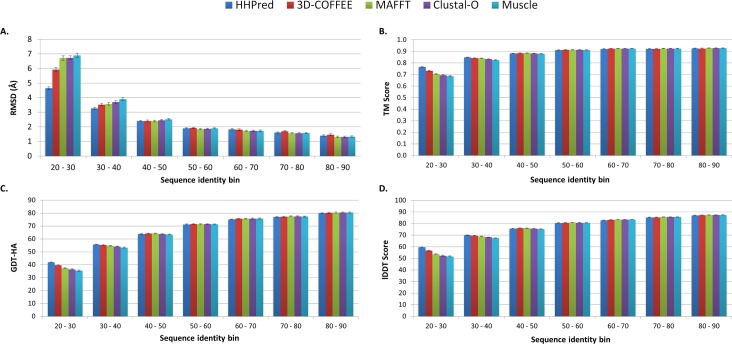
Assessment of the backend scripts of PRIMO. Results shown are the average RMSD values (A) TM scores (B), GDT-HA scores (C) and lDDT scores (D) of models produced for each sequence alignment program in each bin.

The reason for modeling protein targets from the PDB was to be able to evaluate the models produced, by comparing them to known structures. This was done by assessing the RMSD of these structures (Fig 7A). One of the limitations of this approach was that in some cases both the target and template PDB structures were present in different conformations. In some cases, targets and templates had measured RMSD values greater than 20 Å, even at high sequence identity. To account for this, RMSD outliers were removed from each bin before models were evaluated.

In all instances, a similar trend was observed to that shown in the DOPE Z-score assessments. This was not entirely surprising since low DOPE Z-scores (below -0.5) have been previously shown to correspond to lower RMSD values [10]. At lower sequence identity ranges, results were relatively poor and programs such as 3D-Coffee and HHsearch that used structural information performed better than the other alignment programs. From the 50–60% range and above, models had measured RMSD values within 2.0 Å of the target PDBs.

An alternative RMSD measure was also considered by calculating the RMSD value between the template PDB and target PDB, and then subtracting this from the values shown in Fig 7A. This was done as a secondary means of addressing the problem with conformational changes between template and target PDBs. The resulting values (S2 Fig) also make it easier to see the RMSD differences between the different alignment options above the 40–50% sequence identity bin. It was interesting to observe that in the higher sequence identity bins, the alignments produced using 3D-Coffee had an average RMSD value that was greater than those produced by programs that did not take structural information into account, especially in the 70–80% and 80–90% sequence identity bins.

To account for the limitations of calculating RMSD scores, three additional scores were calculated to compare the top models to their respective protein targets. These included two global scores, TM score (Fig 7B) and GDT-HA score (Fig 7C), as well as a calculation local accuracy, the lDDT score (Fig 7D). TM-score provides an indication of accuracy at a protein fold level and is considered a better estimation of model quality than RMSD [35]. GDT scores, such as GDT-HA score are less sensitive than RMSD to deviations that occur in small portions of a model [36]. The TM score results were promising with values above 0.8 in modeling sets above 30% template-target sequence identity (Fig 7B). GDT-HA scores was the strictest measure used, but from the 30–40% bin and upwards these were above 50 (Fig 7C). As a local quality predictor, lDDT score is far less affected by conformational changes than global scores [36]. Our results showed more favorable lDDT scores (Fig 7D) than the GDT-HA.

PRIMO has also been registered to participate in the CAMEO project [4], which allows for independent assessment of the server. Results from this assessment may be viewed at http://cameo3d.org/. Four different modeling options were registered to demonstrate results of using different template identification and alignment approaches, without adding too much additional strain to the PRIMO server. The scores for models produced by PRIMO are comparable to other published servers, such as Phyre2 [16], and are better than the CAMEO baseline, NaiveBlast. Even though PRIMO was not designed to be used as a fully-automated modeling tool, the results from CAMEO will provide valuable feedback for future developments to the server.

### Model refinement results

**Fig 8:**
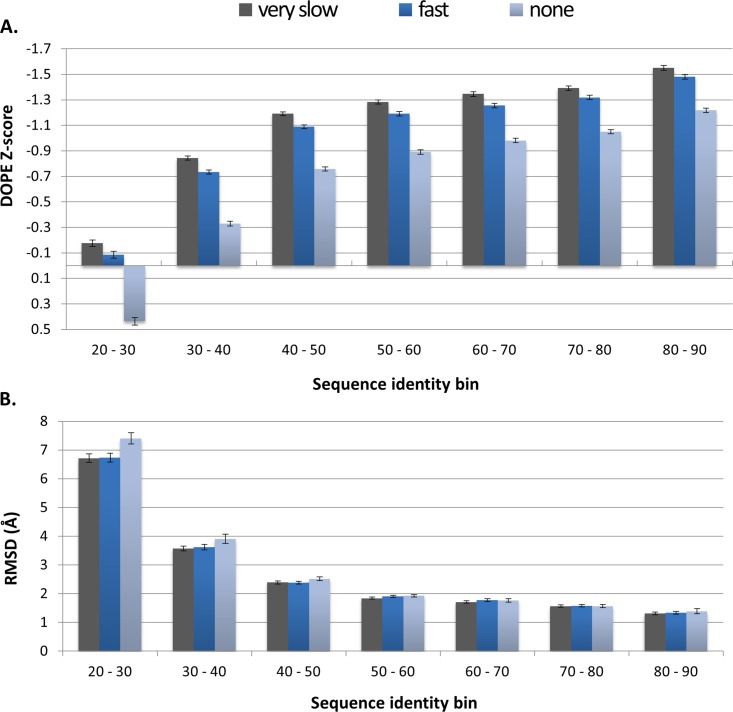
Assessment of model refinement options. DOPE Z-score results (A) and B) RMSD values (B) obtained when testing refinement options provided by MODELLER. Models are divided into sequence identity bins as in Fig 7. Results are shown for refinement options very slow, fast and none.

An additional set of tests were run to quantify the effect of using MODELLER’s different refinement options. The very slow refinement option was selected for the benchmark tests. These results were then supplemented with results using refinement level during modeling set to none and fast (Fig 8). When comparing DOPE Z-scores (Fig 8A), the greatest improvement is seen between no refinement and fast refinement. There is a further improvement when using very slow refinement over fast refinement; however, this difference is far less pronounced. Even more interesting was the RMSD results (Fig 8B). The advantages of using refinement, when modeling are not as clear as with the DOPE Z-score calculations, particularly above 50% sequence identity.

Overall, the benchmark results observed are promising, especially since the PRIMO site was designed with user intervention in mind. By altering parameters, such as using more than one template, manually editing the alignment and increasing the number of models produced, users could easily improve on the results reported here by interacting with the PRIMO pipeline.

### Case studies

To demonstrate the potential ways to use PRIMO, we designed two simple case studies which involved modeling PfHsp70-x and HsTXK proteins.

#### Modeling PfHsp70-x

PfHsp70-x is by no means a challenging target to model and can be considered as a typical protein users might model when using PRIMO. There are templates available with good sequence coverage and sequence identity, making this protein ideal for homology modeling. One of the interesting properties of PfHsp70-x is that, as an Hsp70, it takes on different structural conformations in its different functional states. The PDB contains several structures capturing the different conformations of Hsp70. Thus, homologs of this protein from other organisms can be modeled in these different conformations. This showcases one of the important features of PRIMO; namely the template viewer, which allows users to select and view the conformations of different templates in a similar manner seen when using SWISS-MODEL. This is important because the top models in this case study produced using PRIMO involved multiple template modeling, which should not be done with template structures in different conformations.

The full set of evaluations is summarized in S1(A) Table. As seen in the automated tests, at high sequence identity, there is no clear accuracy gain when using structural alignment programs such as 3D-Coffee, when compared to using MAFFT. Verify3D and DOPE Z-score results indicated that the MAFFT alignment produced slightly better models than those produced using the unaltered 3D-Coffee alignment. This demonstrates the need to test out different modeling approaches, which the PRIMO interface is designed to do.

As part of this case study, we used other online servers to model PfHsp70-x. Our comparison was against the automated features of these servers, but it should be noted that only SWISS-MODEL and HHpred provided a template selection option. Of these, only the SWISS-MODEL interface gave a clear indication of template conformation, which is as important consideration when modeling Hsp70s. As an alignment editing option, HHpred provided a text editor displaying the PIR file to be used by MODELLER. This was nice feature as it gives an indication of PIR file format in addition to allowing users to edit the alignment. It does however, require the user to trim the sequences manually before submitting the job for modeling, which only becomes apparent after the model is returned. The other servers assessed were fully automated, providing to no options beyond the initial input screen. When considering the model evaluation results in S1(A) Table, none of the servers produced poor quality models, which was to be expected, since PfHsp70-x is not a challenging protein to model. What was promising though, was that the models produced by PRIMO were scored more favorably than those produced by the other servers.

Modeling HsTXK. This was a more challenging target to model, as reflected in the evaluation scores S1(B) Table, but it does highlight some interesting features of the different modeling servers. With the exception of the SWISS-MODEL server, all modeling sites returned monomers. SWISS-MODEL returned one of the models as a dimer, as this is the predicted biological assembly, based on template 4xi2, which it used for modeling. In terms of ligand modeling, both SWISS-MODEL and I-TASSER identified ligands in their respective templates, but only I-TASSER included these in the models produced. Neither server provided options to specify which ligands should be included when modeling though. With the PRIMO pipeline, specific inhibitor molecules were selected from each template to be modeled with the protein.

These two case studies were not meant to be a comprehensive assessment of PRIMO compared to other modeling servers, but it was encouraging to see that with this target at least, PRIMO performed well against the other servers assessed for most of the evaluation tools used (S1 Table).

## Conclusions

As a modeling tool, PRIMO aims to provide a middle ground between the lack of control caused by full automation and the difficulty and tedious nature of writing scripts and using modeling programs though the command line. The site can identify templates using both HHsearch and BLAST, perform sequence alignments with one of five different alignment options, and perform homology modeling using MODELLER. PRIMO incorporates a job history system which allows quick and easy navigation among the different steps of a specific modeling job, as well as navigation between different jobs. With this, users can perform several modeling jobs in parallel, while also being able to go back and alter modeling parameters to achieve the best results.

The PRIMO pipeline allows users of varying levels of experience to perform homology modeling interactively and reliably. While this ‘hands on’ approach to modeling is largely encouraged, we still aim to ensure that the automated modeling features of this pipeline are as accurate as possible. The accuracy tests reported here demonstrate that the automation of these algorithms can be done with promising accuracy down to 40% sequence identity, which is where comparative modeling is known to reach its limits [6]. The accuracy of PRIMO’s automated modeling capabilities are continuously being assessed by CAMEO.

As a web interface, PRIMO is platform independent and requires no personal computing power. The site currently provides a means for modeling protein monomers using one or more templates and provides functionality to allow protein-ligand complex modeling. Unlike other servers, PRIMO allows users to select specific ligands and ions to be included in the modeling process.

Since PRIMO works via communication with the JMS, adding to the features and functionality of this pipeline can be achieved by simply adding new tools to the JMS. In future we will add more options for template identification, sequence alignment and model evaluation where possible. For now, PRIMO provides a quick and easy way to perform homology modeling, while allowing users to make alterations and improvements to their modeling jobs.

In summary, PRIMO prides itself on providing flexibility during the modeling process, giving users the ability to exercise a certain degree of control over their modeling jobs. It allows users to edit parameters and rerun jobs, while the navigational bar and job history features allow users to attempt multiple modeling approaches in tandem to optimize their modeling results. The site incorporates a user friendly design, which is simple to use, yet robust. The site is intuitive to use, with all options being easy to find and test out; which adds to the educational value of the site, as users gain hand-on experience with homology modeling. Users can adjust parameters and see the effect on the models produced. Apart from the model evaluation options provided by the site, PRIMO links to various other evaluation servers, which inexperienced users should find helpful. In addition to this, tips and tricks are provided in the loading screen to give novice users suggestions as to how they may improve their modeling runs.

Future development will focus on providing more features, such as protein complexes, including modeling of biological assemblies specified within template PDB files. PRIMO encourages user involvement in the homology modeling process and as such we shall also aim to provide additional options for each of the stages.

The PRIMO webserver may be accessed freely for academic use at https://primo.rubi.ru.ac.za

## Supporting Information

S1 Fig Sequence identity box plots. The box plots show measured target-template sequence identity for all modeling sets, divided into their sequence identity bins and alignment program used, as measured based on the PIR file used for modeling. These are shown for all models produced before the filtering step (Fig 3B).
(TIF)

S2 Fig RMSD_Diff_ results obtained from testing the backend scripts of PRIMO. Results are shown for the data set used in Fig 7. The RMSD_Diff_ value was calculated by subtracting the RMSD value between the template PDB target PDB from the RMSD value measured between the top model and the target PDB.
(TIF)

S1 Table Model evaluation results for modeling using PRIMO and various other modeling servers. Proteins PfHsp70-x (A) and HsTXK (B) were modeled and evaluated. Models for each server are shown along with quality scores measured by ProSA, Verify3D, the QMEAN server, PROCHECK and DOPE Z-score. The PROCHECK results are sub-divided as follows: Fav–Residues in most favored regions; Add—Residues in additional allowed regions; Gen—Residues in generously allowed regions; Dis. Residues in disallowed regions.
(DOCX)
