# Supplementary figures and images for: PRIMO: An Interactive Homology Modeling Pipeline
Source: PLoS One. 2016 Nov 17;11(11):e0166698. doi: 10.1371/journal.pone.0166698 (PMC5113968; doi:10.1371/journal.pone.0166698)

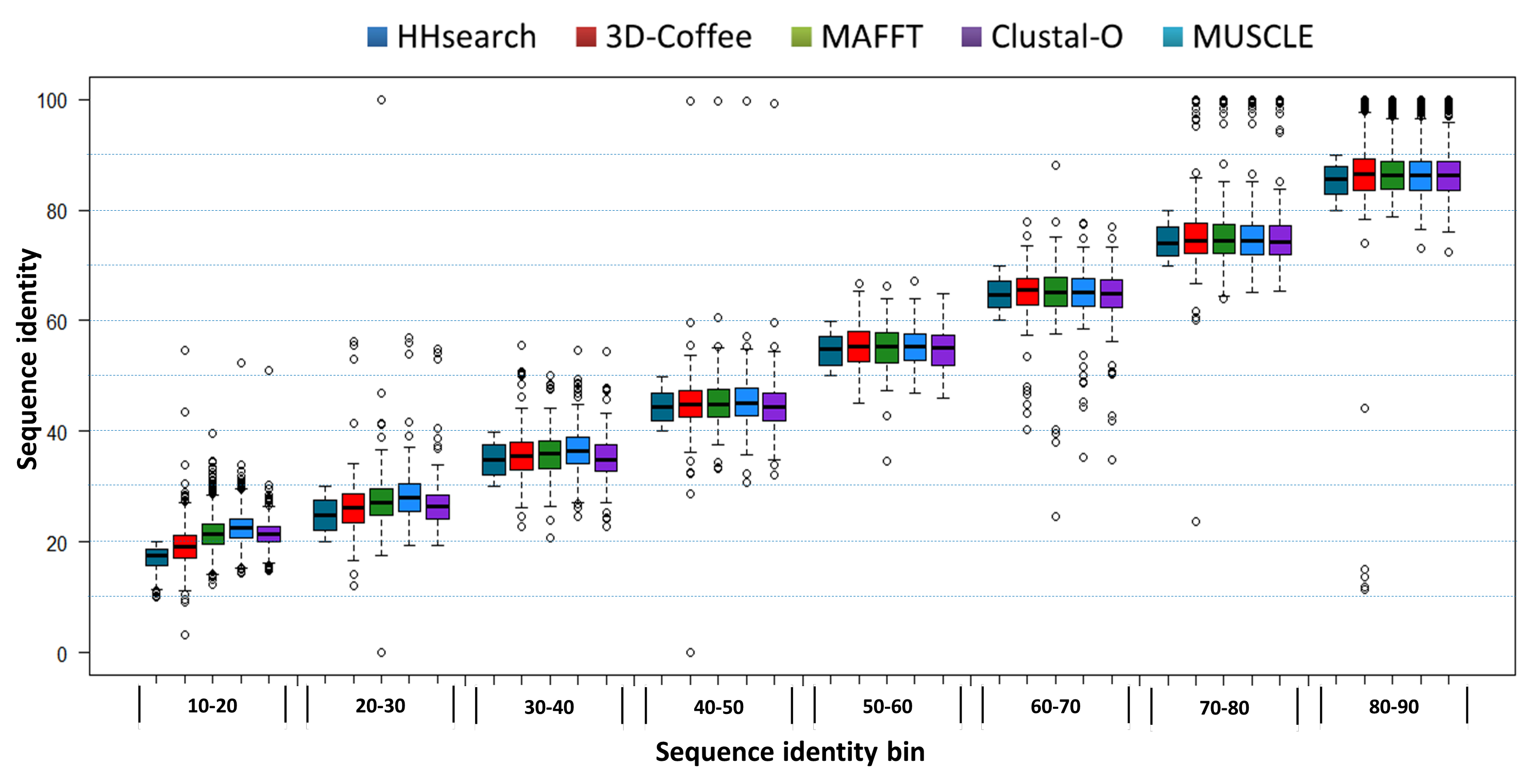

Supplement: S1 Fig — The box plots show measured target-template sequence identity for all modeling sets, divided into their sequence identity bins and alignment program used, as measured based on the PIR file used for modeling. These are shown for all models produced before the filtering step (Fig 3B). (TIF) [file pone.0166698.s001.TIF]

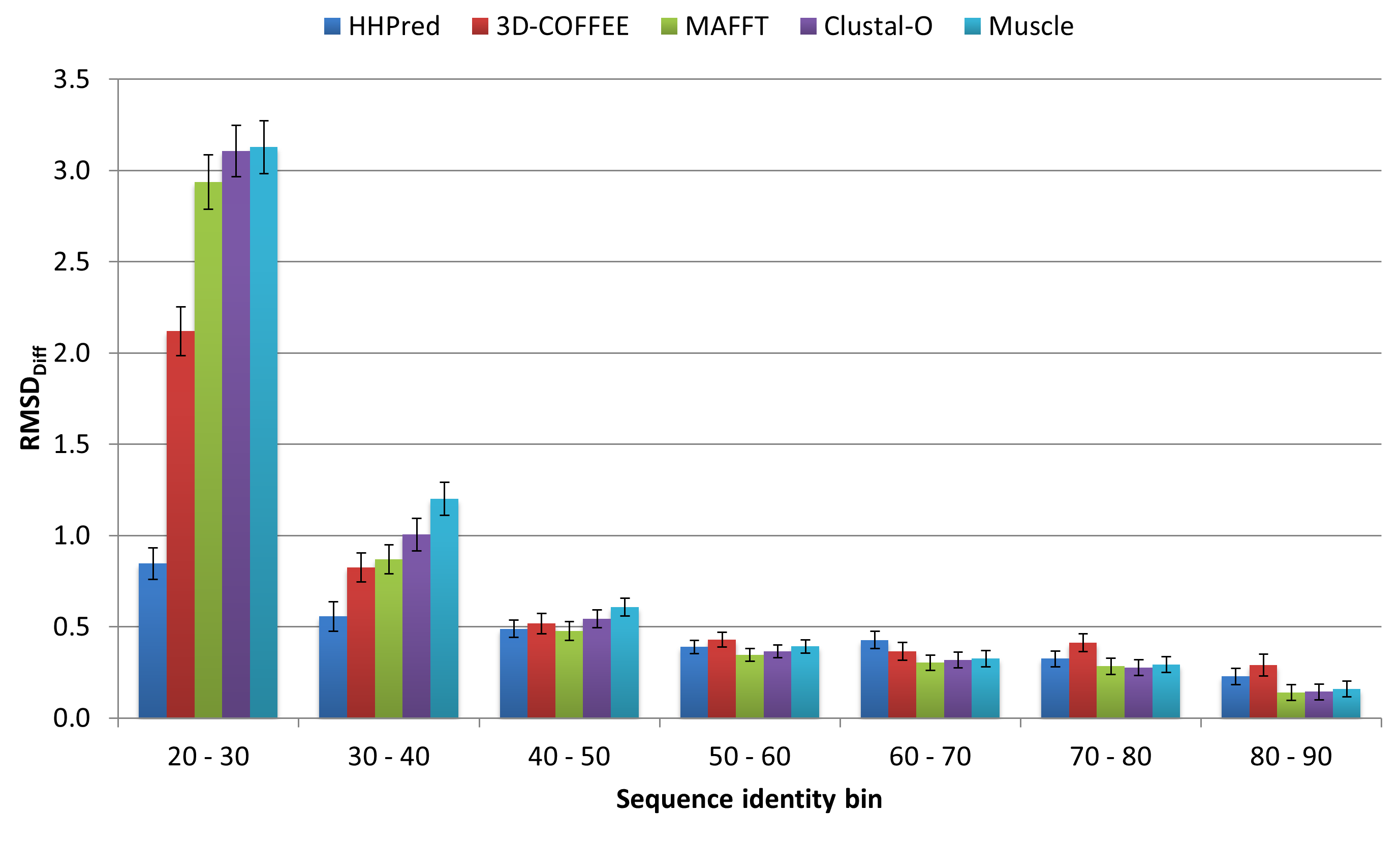

Supplement: S2 Fig — Results are shown for the data set used in Fig 7. The RMSDDiff value was calculated by subtracting the RMSD value between the template PDB target PDB from the RMSD value measured between the top model and the target PDB. (TIF) [file pone.0166698.s002.TIF]
