# Supplementary material for: PRIMO: An Interactive Homology Modeling Pipeline
Source: PLoS One. 2016 Nov 17;11(11):e0166698. doi: 10.1371/journal.pone.0166698 (PMC5113968; doi:10.1371/journal.pone.0166698)
Supplement: S1 Table — Proteins PfHsp70-x (A) and HsTXK (B) were modeled and evaluated. Models for each server are shown along with quality scores measured by ProSA, Verify3D, the QMEAN server, PROCHECK and DOPE Z-score. The PROCHECK results are sub-divided as follows: Fav–Residues in most favored regions; Add—Residues in additional allowed regions; Gen—Residues in generously allowed regions; Dis. Residues in disallowed regions. (DOCX) [file pone.0166698.s003.docx]

| 1. **PfHsp70-x** | | | | | | | | | |
| --- | --- | --- | --- | --- | --- | --- | --- | --- | --- |
|  | **ProSA** | **Verify3D** | **QMEAN Server** | | **PROCHECK** | | | | **MODELLER** |
| **Model** | **Z-score** | **% Residues with 3D-1D score >= 0.2** | **QMEAN** | **Z-score** | **Fav.** | **Add.** | **Gen.** | **Dis.** | **DOPE Z-score** |
|  |  |  |  |  |  |  |  |  |  |
| PRIMO_3DCs: | -11.87 | 92.8% | 0.632 | -1.44 | 93.8% | 5.1% | 0.4% | 0.7% | -1.33 |
| PRIMO_3DC_Manedit: | -11.91 | 98.0% | 0.631 | -1.47 | 93.9% | 5.5% | 0.4% | 0.2% | -1.35 |
| PRIMO_MAFFT: | -11.84 | 97.9% | 0.636 | -1.40 | 93.6% | 6.2% | 0.2% | 0.0% | -1.35 |
| PRIMO long model: | -11.67 | 98.9% | 0.638 | -1.38 | 93.2% | 6.4% | 0.4% | 0.0% | -1.24 |
|  |  |  |  |  |  |  |  |  |  |
| Phyre2: | -11.09 | 91.7% | 0.604 | -1.76 | 89.8% | 8.5% | 1.6% | 0.2% | -0.86 |
|  |  |  |  |  |  |  |  |  |  |
| SWISS-MODEL 1: | -11.60 | 96.9% | 0.599 | -1.82 | 86.6% | 11.0% | 1.3% | 1.1% | -1.14 |
| SWISS-MODEL 2: | -11.57 | 95.3% | 0.618 | -1.61 | 86.2% | 11.0% | 1.5% | 1.3% | -1.19 |
|  |  |  |  |  |  |  |  |  |  |
| I-Tasser_model1: | -11.14 | 96.6% | 0.543 | -2.44 | 72.9% | 18.1% | 4.8% | 4.1% | -1.13 |
| I-Tasser_model2: | -11.40 | 98.6% | 0.503 | -2.87 | 73.6% | 16.9% | 5.4% | 4.1% | -1.13 |
|  |  |  |  |  |  |  |  |  |  |
| HHpred_5e84_trim: | -11.78 | 96.7% | 0.617 | -1.62 | 93.0% | 5.7% | 0.9% | 0.4% | -1.08 |
|  |  |  |  |  |  |  |  |  |  |
| modbase-model_3d2fA: | -10.85 | 87.2% | 0.553 | -2.33 | 92.0% | 6.5% | 1.3% | 0.2% | -0.37 |
| modbase-model_3dobA: | -7.78 | 86.8% | 0.584 | -1.98 | 90.0% | 8.4% | 0.7% | 0.9% | -0.31 |

| **B. HsTXK** | | | | | | | | | |
| --- | --- | --- | --- | --- | --- | --- | --- | --- | --- |
|  | **ProSA** | **Verify3D** | **QMEAN Server** | | **PROCHECK** | | | | **MODELLER** |
| **Model** | **Z-score** | **% Residues with 3D-1D score >= 0.2** | **QMEAN** | **Z-score** | **Fav.** | **Add.** | **Gen.** | **Dis.** | **DOPE Z-score** |
|  |  |  |  |  |  |  |  |  |  |
| PRIMO_4ot5: | -7.43 | 91.63% | 0.798 | 0.3 | 92.3% | 6.4% | 0.9% | 0.4% | -1.25 |
| PRIMO_1opt: | -7.99 | 93.42% | 0.655 | -1.33 | 88.6% | 9.1% | 1.8% | 0.5% | -0.08 |
|  |  |  |  |  |  |  |  |  |  |
| Phyre2: | -7.4 | 73% | 0.635 | -1.56 | 76.1% | 18.5% | 2.8% | 2.5% | 2.08 |
|  |  |  |  |  |  |  |  |  |  |
| SWISS-MODEL BA: | -7.39 | 85% | 0.732 | -0.41 | 87.8% | 9.9% | 1.5% | 0.8% | -0.33 |
| SWISS-MODEL Mono: | -7.71 | 90.89% | 0.75 | -0.2 | 88.3% | 9.9% | 1.3% | 0.5% | -0.93 |
|  |  |  |  |  |  |  |  |  |  |
| I-Tasser_model1: | -8.24 | 85.23% | 0.667 | -1.17 | 76.7% | 18.0% | 4.1% | 1.3% | -0.74 |
| I-Tasser_model2: | -6.8 | 76.82% | 0.651 | -1.36 | 78.%5 | 17.2% | 2.5% | 1.8% | -0.17 |
|  |  |  |  |  |  |  |  |  |  |
| HHpred_4xi2_trim: | -7.11 | 75.73% | 0.708 | -0.69 | 91.2% | 7.8% | 0.5% | 0.5% | 0.00 |
|  |  |  |  |  |  |  |  |  |  |
| modbase-model_01: | -7.8 | 88.38% | 0.702 | -0.77 | 88.8% | 9.4% | 1.3% | 0.5% | -0.42 |
